# Autistic Individuals Are Flexible with Physical and Emotion Gradable Adjectives

**DOI:** 10.3390/bs16020297

**Published:** 2026-02-19

**Authors:** Leo Evans, Peter DeVilliers, Letitia Naigles

**Affiliations:** 1 Boys Town National Research Hospital, Omaha, NE 68131, USA; lee.evans@boystown.org; 2 Department of Psychology, Smith College, Northhampton, MA 01063, USA; pdevilli@smith.edu; 3 Department of Psychological Sciences, University of Connecticut, Storrs, CT 06269, USA

**Keywords:** lexicon, gradable adjectives, emotions, adolescence, autism

## Abstract

Gradable adjectives (long, happy) differ from absolute adjectives (spotted) in that they are dependent on context and speaker/listener perspective for their interpretation. Such context sensitivity may present challenges for individuals with autism spectrum disorder (ASD); however, this has never been investigated for these linguistic elements. In the current study, we asked adolescents with ASD or typical development (TD), who were part of a larger longitudinal study in which autistic characteristics, nonverbal cognition (NVIQ), and standardized language were also assessed, to sort pictures whose properties were either gradable or absolute. Adolescents sorted pictures on two occasions. In the second sorting, we manipulated the context by adding images representing one end of the scale to induce a shift in interpretation. Contrary to prediction, both groups demonstrated sensitivity to the context-specific properties by shifting their cutoffs of what counted as ‘long’ or ‘happy’ when the array was changed. Whereas NVIQ correlated positively with physical property shifts for the TD group, language measures correlated negatively with emotion property shifts for the ASD group. Autistic characteristics were not related to shift patterns in either group. Adolescents with autism are clearly able to take context into account when interpreting gradable adjectives; however, those with better language seem more focused on maintaining their cutoffs more than shifting them.

## 1. Introduction

Gradable adjectives (*long*, *happy*) are distinguished from absolute adjectives (*spotted*) in requiring sensitivity to context and shifting perspectives for their interpretation (Kennedy, 2007); nonetheless, typically developing children as young as four years of age appear to have acquired the basics of the absolute/gradable adjective distinction (Syrett et al., 2006). Individuals with autism, whose characteristic profile includes challenges with understanding pragmatic contexts and set shifting (Demetriou et al., 2019; Naigles & Chin, 2015), may have more difficulty with this contrast.^1^ Moreover, mastering gradable adjectives that reference emotions, such as *happy* or *sad*, may pose additional challenges because of documented inconsistencies in emotion recognition (e.g., Golan et al., 2017). Research on language in individuals with autism has not yet focused on the absolute/gradable adjectives contrast; the current study fills this gap by directly assessing the understanding of gradable adjectives, both physical and emotion, in adolescents with ASD, and comparing this to their typically developing peers.

### 1.1. Absolute vs. Gradable Adjectives and Typical Development

In Indo-European languages such as English, adjectives can be distinguished as *absolute* or *relative/gradable*, whereby absolute adjectives apply to nominal arguments in an all-or-nothing fashion, whereas relative adjectives apply to nominal arguments by degrees, which are ordered with respect to some physical or abstract dimension (Kennedy, 2007). For example, the absolute adjectives *spotted* or *striped* can only be applied to nominal arguments (i.e., objects) that have at least one spot or stripe, and it is equally appropriate to use these terms for those objects that boast many stripes or spots, as for those objects that show just one stripe or spot. The number, size, or other kind of variability of spots or stripes does not change the appropriateness of using these adjectives with reference to the objects. In contrast, the relative or gradable adjectives *long*, *short*, *happy* and *angry* can be applied to any nominal arguments (objects, animates), depending on the surrounding context and/or speaker orientation. For example, caterpillars may be considered long compared to ladybugs, but short compared to garter snakes; moreover, it is appropriate to say that some caterpillars are *longer* than others. Emotions, too, vary by context and speaker orientation: fans of the runner-up in American baseball’s fall classic may be happy that their team played in the World Series (and be happier than fans of teams that did not); but as their team did not win enough games to be champions, they may be less happy than fans of the champion team. Similarly, it is appropriate to say that these fans were *happier* at the beginning of the World Series than at its conclusion.

Research with neurotypical adults on the absolute/gradable adjective contrast has primarily focused on their intuitions about and processing of physical adjectives, demonstrating both semantic and pragmatic contributions. For example, Foushee and Srinivasan (2017) reported that adults rated disagreement to be much more likely when speakers assign relative adjectives to objects, compared with absolute adjectives. Moreover, Xiang et al. (2022) gave adults both a truth conditional (i.e., semantic) task (Is this striped/long?) and a probability (i.e., pragmatic) task (How striped/long is this object?) and found that their participants distinguished absolute and gradable adjectives in both tasks, showing that these adjectives include both semantic and pragmatic components. Finally, in a precursor to the current study, Britton et al. (2017) asked adults to sort nine pictures of physical objects or emotional faces that varied systematically in degree of length, emotional valence, or number of stripes/spots, placing the, e.g., long/striped/happy items in the designated box. Cutoffs of what counted as long/striped/happy were then determined for each set. On a subsequent day, four additional extreme pictures were added to each set (i.e., equivalent to the longest pencils, most sparsely striped umbrellas, happiest faces); the adults were asked to sort the 13 pictures, and cutoffs were again established. Britton et al. (2017) demonstrated that for the gradable adjectives, adults consistently shifted their cutoffs from day 1 to day 2, moving them closer to the extreme end of the set; no changes in cutoff were observed for the absolute adjectives.

This absolute vs. gradable adjective contrast also seems well within the grasp of typically developing children. Foushee and Srinivasan (2017) found that nine-year-olds made similar ratings of speakers’ agreement (on absolute adjectives) and disagreement (on gradable adjectives) as had their adult participants. More impressively, Barner and Snedeker (2008) demonstrated that three- and four-year-olds were sensitive to context when sorting objects by physical adjectives such as *tall* and *short*, placing more objects in a ‘tall’ category when they were part of an array that included shorter objects. Syrett et al. (2006) demonstrated that preschoolers were sensitive to context for selecting objects designated by gradable adjectives (‘pick out the tall one’ yielding the taller of two objects) but less swayed by context when selecting objects designated by absolute adjectives (pick out the ‘spotted’ one yielding either the more or less densely spotted object). Syrett et al. (2010) replicated these latter findings with a larger array of absolute and gradable adjectives, now including *sad* and *happy* in the latter set; however, they did not report the findings from the emotion adjectives separately. And Britton et al. (2017) gave the same sorting task (nine items per set on day 1 and 13 items per set on day 2) to 4-year-olds as they had given adults and found similar shifts in cutoffs on day 2 for the gradable physical and emotion adjectives, but not for the absolute adjectives. In sum, the absolute/gradable adjective contrast, and its dependency on context (including speaker orientation, focus, and/or agreement), seems well-attested in English-speaking adults and children as young as 3–4 years of age.

### 1.2. The Absolute/Gradable Adjective Contrast in Autism

No published work that we know of has considered how individuals with autism might treat the absolute/gradable adjective contrast, either with physical adjectives or emotion adjectives. This investigation is the purpose of the current study. Given several reported characteristics of the autism profile (Demetriou et al., 2019; Miller et al., 2015), one might conjecture that individuals with autism might have difficulty with this contrast and might have more difficulty with emotion adjectives than with physical adjectives. The contextual aspects of gradable adjectives may require listeners to shift their own perspectives when generating interpretations; e.g., recognizing that what they perceive to be a ‘low’ stool or a ‘short’ walk out to the car may be perceived as ‘high’ or ‘long’ to younger humans or smaller pets. Such perspective-taking or set-shifting is documented to be challenging for individuals diagnosed with autism, who in many cases see the world in absolutes. (American Psychiatric Association, 2013; Demetriou et al., 2019; Miller et al., 2015). For example, autistic individuals make more mistakes on the STROOP task compared to neurotypical individuals, when shifting from reporting colors to reporting words and vice versa (see review in Yeung et al., 2024). Moreover, within the language domain, pragmatic challenges are among the most consistently reported in individuals with autism, including difficulties with providing sufficient information within conversation and/or narratives to orient addressees/listeners (Andrés-Roqueta & Katsos, 2017; Naigles & Chin, 2015; Suh et al., 2014).

Challenges with emotion recognition are also well-documented within the ASD profile. For example, Taylor et al. (2015)—see also (Golan et al., 2017; Löytömäki et al., 2020)—asked school-age children with ASD and mental-age-matched TD children to match photos or cartoons of emotional faces with the voices conveying those emotions and reported significantly poorer performance in the ASD group. Moreover, analyses of the narratives and conversations of individuals on the autism spectrum have consistently found fewer emotion adjectives or references to emotional sentiments (e.g., Chojnicka & Wawer, 2020; Suh et al., 2014; see Martínez-Tomás et al., 2025 for a review).

In sum, we might predict that autistic individuals would have difficulty with distinguishing absolute and gradable adjectives and particular difficulties (i.e., less context sensitivity) pertaining to emotion adjectives. However, some studies have demonstrated that higher-verbal children or adults with autism are just as accurate with matching simple emotion words to faces as language-matched controls, pointing to a role for word meaning (and not just emotion recognition) in this process (Grossman et al., 2000; Icht et al., 2021). Emotion recognition, with and without linguistic prompts, is also frequently the focus of intervention studies, several of which have reported significant improvements post-treatment (e.g., Baron-Cohen et al., 2009; Golan et al., 2017). Longitudinal studies have further documented improvements in most components of executive functions, including set-shifting, of equivalent magnitude in children with ASD and TD children. Moreover, the acute attention to visual–spatial detail frequently manifested by individuals with autism (Mottron et al., 2006) has the potential to actually enhance context sensitivity with physical gradable adjectives because the individuals might weight the adjusted variability in spatial dimensions more heavily. Thus, our study of gradable physical and emotion adjectives, which uses the Britton et al. (2017) task and includes both higher and lower verbal adolescents and young adults with autism, might show the expected challenges, or surprising strengths. To further explore possible bases for good vs. poor performance, we will examine relationships with the participants’ autistic characteristics, cognitive levels, and language levels. Because reluctance or proclivity to shift cutoffs might relate in general to autistic characteristics such as restricted and repetitive behaviors, we hypothesize that both physical and emotion gradable adjective cutoff shifts will relate to ADOS scores. In contrast, we hypothesize that proclivity to shift cutoffs with physical gradable adjectives might relate most strongly to spatial acuity or spatial reasoning, and we hypothesize that proclivity to shift cutoffs with emotion gradable adjectives might relate most strongly to linguistic sophistication.

## 2. Materials and Methods

### 2.1. Participants

Participants were a subset of an ongoing longitudinal study’s sample, tested about 15 years after study onset (‘Onset’). Children on the spectrum were initially recruited, through ASD service providers in the area, to be within six months of their diagnosis and using little to no phrase speech. TD children were recruited locally, via birth announcements, flyers, and community word of mouth, to have similar spoken language characteristics to the autism sample. Consequently, at the Onset visit, the TD children averaged 1.5 years of age and the children on the spectrum averaged one year older. Every participant’s diagnostic status was confirmed via administration of the Autism Diagnostic Observation Schedule—Generic (ADOS-G; Lord et al., 2000), and the two groups were matched on expressive language, operationalized as raw scores on the expressive language subscale of the Mullen Scales of Early Learning (MSEL; Mullen, 1995). Details of our child participants at their Onset visit are presented in Table 1.

The current study was conducted at the most recent of the study’s visits (‘Outcome’). The subset who was seen at the Outcome visit included 24 TD adolescents and 23 autistic adolescents; however, four TD adolescents were excluded due to loss of data, and five autistic adolescents were excluded due to inability to understand and complete the task. Thus, the final sample included 20 TD adolescents (four girls, 16 boys) and 18 autistic adolescents (two girls, 16 boys). Due to the timing of the Outcome visit’s data collection, the groups did not differ statistically in age, though the ASD group was numerically older. Moreover, they now differed in language as well as NVIQ, and continued to differ in autism symptomatology, as shown in Table 2.

### 2.2. Overall Procedure

Testing was conducted in the participants’ homes at all visits. Measures at each timepoint were administered in two sessions, at most two weeks apart.

### 2.3. Standardized Test Measures

Participants’ language, NVIQ, and autism symptomatology were assessed with age-appropriate measures at all timepoints. For details about each measure, see Table 3.

### 2.4. Gradable Adjective Task

#### 2.4.1. Materials

**Figure 1:**
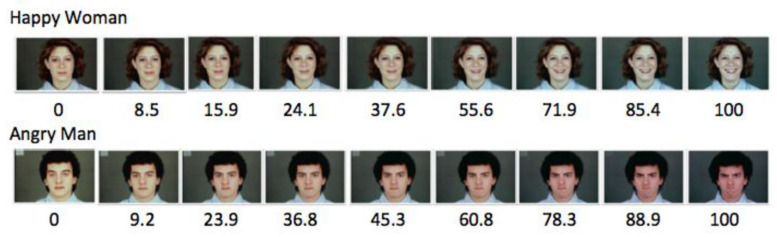
Examples of the nine-picture emotion sequences created by morphing the neutral face (rated 0) with the most intense pictured emotion (rated 100). Numbers indicate the mean ratings across 24 adults of the intensity of the emotion depicted in each picture. For each of the six emotion picture sets in the study, the ratings of perceived intensity of emotion were approximately linear.

There were nine sets of stimulus items, including two control sets of non-gradable physical dimensions, one set of gradable physical dimensions, and six sets of gradable emotion faces. The two control sets of stimuli depicted striped umbrellas and spotted dogs. For the ‘day 1’ materials, five of the items depicted umbrellas with varying numbers of stripes, and four items depicted umbrellas with no stripes; for the ‘day 2’ materials, four additional umbrellas with no stripes were included. Similarly, five ‘day 1’ items depicted dogs with varying numbers and sizes of spots, and four items depicted dogs with no spots. For the ‘day 2’ materials, four additional dogs with no spots were included. The gradable physical set included nine pencils of varying lengths on day 1; for day 2, four pencils were added that were the length of the longest pencil in the day 1 set. Finally, the six emotion sets included the faces of a man and a woman, each morphing across nine pictures from happy to neutral, from sad to neutral, and from angry to neutral (see Figure 1). Ratings of the pictures by 24 adults produced roughly linear 9-picture sequences scaled for the intensity of the expression of each emotion. For the day 2 materials, four additional pictures were added to each set, depicting faces that were the highest level of extremely happy, sad, or angry depicted on day 1.

The control sets were included to provide a potential comparison of how absolute adjectives would be sorted differently in this task; they also could reveal whether individuals simply deployed a strategy of assigning half of the items to the ‘spotted’/’happy’ box each day (in which case the sorts for both control and gradable adjectives would change when the set size increased).

#### 2.4.2. Procedure

On day 1, participants were shown one stimulus set at a time and told to look through the pictures or across the set of pencils. The examiner shuffled the cards or pencils and then set them out in a random fashion in front of the participant. Each participant received the same stimuli sets in the same order: happy man, angry woman, striped umbrella, sad man, happy woman, long pencil, angry man, spotted dog, sad woman. After perusing all items in a given set, they were given a box and asked to put all of the, e.g., ‘happy’ items in that box. Although each participant accomplished this sorting quickly, each was allowed as much time as they needed. Non-committal feedback (‘thanks!’) was provided. On day 2, participants were reminded of the items (‘Remember how these faces varied from being happy to being neutral?’) and informed that there were now more items in each set but that the task was the same.

#### 2.4.3. Coding

The experimenter recorded which of the stimuli the participants put in the box, and which they kept out of the box, and used this to determine the cutoff value for each adjective.

Cutoff values for the absolute adjectives were coded for their predetermined correct answers, as the dogs were either spotted or not spotted, and the umbrellas were either striped or not striped. There were five striped umbrellas, and five spotted dogs shown, and in order to be correct, the participants must have answered with the five spotted/striped pictures in each category.

The items depicting gradable adjectives were numbered on their reverse sides, sequentially from neutral face (shortest pencil) to happiest/saddest/angriest face (longest pencil). Cutoff values for these sets were coded as the lowest number the participant placed in the box; that is, the lowest value to which the participant assigned the given characteristic (i.e., ‘happy’, ‘sad’, ‘long’, etc.). For example, a participant who placed the angry faces items numbered 4,5,6,7,8,9 in the box, leaving the items numbered 1,2,3 out of the box, would be coded with a cutoff value of 4; i.e., the lowest value assigned to the emotion considered to be angry was ‘4’. Occasionally, participants omitted one or more items from the sequence for their ‘in box’ placements. For example, one participant placed the angry faces items numbered 3,4,5,7,8,9 in the box, leaving the items numbered 1,2,6 out of the box. Our coding allowed for just one item out of sequence (the 6); therefore, this participant’s cutoff for angry faces would be 3. The groups did not differ with respect to placing just one item out of sequence: within the TD group, 21.67% of trials included just one item out of sequence; within the ASD group, this was 29.6% of trials; *t*(36) = 1.4, *p* = 0.169. On even rarer occasions, participants ‘misplaced’ two or more items from the sequence; these trials (2 for the TD group, 5 for the ASD group) were not included in the analyses.

### 2.5. Analysis Plan

To address our first research question, whether the addition of more extreme exemplars on the second day influenced participants’ sorting behavior, we fit three separate linear mixed-effects models using the lmer function from the lme4 package in R (Bates et al., 2015; R Core Team, 2024). Each model corresponded to a different adjective type: fixed adjectives, gradable physical adjectives, and emotion adjectives. The dependent variable was the cutoff value assigned during the sorting task. The fixed effects included Day (Day 1 vs. Day 2), Group (e.g., Control vs. Experimental), and their interaction (Day × Group) to test whether shifts differed between groups. Random intercepts were included to account for repeated measures within participants. The model equation for each adjective type was specified as follows:

Cutoff*ij* = *β*0 + *β*1Day*ij* + *β*2Group*j* + *β*3(Day*ij* × Group*j*) + *uj* + *ϵij*.

To address our second research question, whether participants’ cutoff shift patterns were associated with individual differences in autistic traits, cognitive ability, and language skills, a series of regression analyses were conducted in the R environment. Each regression model tested whether the relationship between the predictor and the outcome variable (e.g., Pencil Cutoff Shift or Emotion Cutoff Shift) was moderated by diagnostic group (TD or ASD). The general moderation equation was as follows:

Outcome = *β*0 + *β*1Predictor + *β*2Group + *β*3(Predictor × Group) + *ϵ*.

Per our hypotheses, regression analyses were conducted to examine how participants’ cutoff shift patterns for both pencil and emotion stimuli might relate to the ADOS, how their cutoff shift patterns for the pencil stimuli might relate to the DAS-II, and how their cutoff shift patterns for the emotion stimuli might relate to the CELF-5. For the DAS-II, main analysis regression models were fit for the composite scales of Nonverbal Reasoning Ability (e.g., Matrices, Pattern Construction), Spatial Ability (e.g., Recall of Designs, and Pattern Construction) and the Special Nonverbal Composite (e.g., Matrices, Sequential and Qualitative Reasoning). Given the overlap in subtests comprising the composites, additional follow up regressions were run on each subtest individually. For the CELF-5, main analysis regression models were fit for the Expressive Language Index as well as the total Raw Score children received. Raw scores were used to estimate actual ability without taking age into consideration. Follow-up models were fit for individual subtests.

Diagnostic checks indicated that residuals from ordinary least squares models violated normality assumptions. To address this, robust regression using MM-estimators was implemented via the lmrob() function in the *robustbase* package R version 4.4.2. This approach reduces the influence of outliers and non-normality, providing more reliable estimates of interaction effects. Because analyses were exploratory (due to small sample size) and involved multiple tests across three conceptual areas (autistic traits, nonverbal cognitive measures, and language measures), False Discovery Rate (FDR) correction was applied to ADOS models and language/cognitive models separately using the Benjamini–Hochberg procedure to control for the large number of interactions. Significant interaction terms indicate that the relationship between the predictor and outcome differs by group.

## 3. Results

Our first research question asked whether the addition of more extreme exemplars on the second day shifted participants’ perceptions (i.e., sorting) of the items, whether this occurred for both gradable physical adjectives and emotion adjectives, and whether the ‘day 2’ shifts differed between groups. The mean (SD) cutoff values for each day and item type are presented in Table 4.

First, to verify that day 1/day 2 cutoff shifts did not occur for the control non-gradable adjectives (striped, spotted), we fit a linear mixed-effects model predicting non-gradable scores from Day (1 vs. 2), Group (ASD vs. TD), and their interaction, with a random intercept for participant to account for repeated measures. As expected, there were no significant effects of Day (*p* = 1.00), Group (*p* = 0.94), or their interaction (*p* = 1.00). The outcome showed minimal variability, with only two ASD participants differing across sessions, confirming that this measure remained stable across groups and timepoints.

We next examined whether registration of pencil length as ‘long’ varied as a function of Day (day 1 vs. day 2) and Group (ASD vs. TD), again using a linear mixed-effects model with a random intercept for participant (ID). The initial model included fixed effects of Day and Group. Adding the interaction term did not significantly improve model fit, χ^2^(1) = 1.05, *p* = 0.30. Therefore, we interpret results from the more parsimonious model without the interaction. In this model, there was a significant main effect of Day, *b* = 1.00, *SE* = 0.20, *t*(38) = 4.89, *p* < 0.001, indicating that the cutoff values for deeming pencils to be ‘long’ were significantly longer on day 2 than day 1. There was no main effect of Group (*p* = 0.93), suggesting that overall conception of pencil length did not differ between ASD and TD participants.

**Figure 2:**
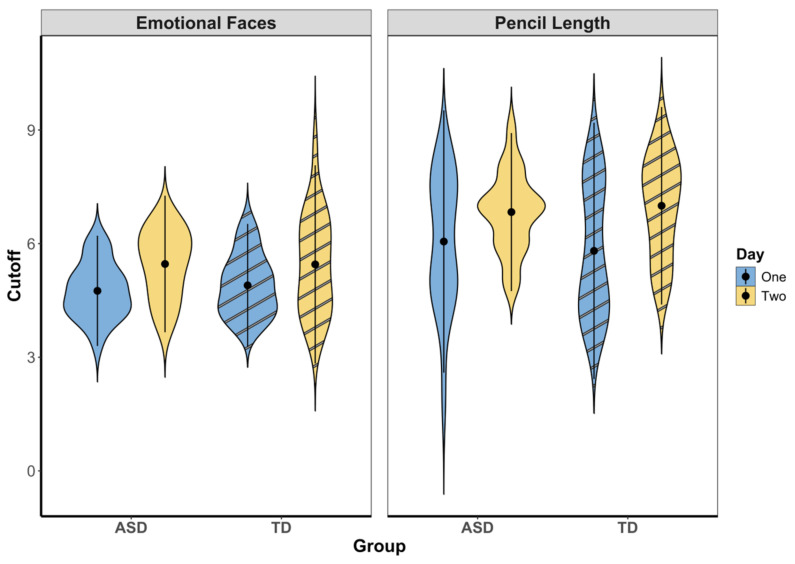
Violin plots showing overall emotional faces or pencil length cutoff scores (y-axis) by group (ASD and TD; x-axis), for day 1 and day 2. *Note.* Cutoff score shift differed by day and did not differ by group.

We then examined whether cutoff points for emotional faces varied as a function of Day (day 1 vs. day 2) and Group (ASD vs. TD) using a linear mixed-effects model with a random intercept for participant (ID). The initial model included fixed effects of Day and Group. A second model adding the interaction term did not significantly improve model fit, χ^2^(1) = 0.37, *p* = 0.54. Therefore, we interpret results from the more parsimonious first model. There was a significant main effect of Day, *b* = 0.63, *SE* = 0.13, *t*(38) = 4.85, *p* < 0.001, indicating that cutoff points for emotions were higher on day 2 than day 1. There was no main effect of Group (*p* = 0.82), suggesting that overall cutoff points did not differ between ASD and TD groups. This pattern indicates that while thresholds for the emotion adjectives of ‘happy’, ‘sad’, and ‘angry’ increased across sessions, this change was consistent across groups, suggesting no differential change in emotional registration between ASD and TD participants; see Figure 2.

While both TD and ASD groups shifted their cutoff values for the emotion items overall, these shifts were by no means ubiquitous across participants and items. Of the six emotion items presented, with respect to the number of cutoff shifts made, all of the participants on the autism spectrum shifted cutoff values for at least one item, as did all but one of the TD participants; however, only one participant shifted cutoff values for all emotion items. The ASD group shifted their cutoffs on an average of 3.06 (SD = 1.47; range 1–6) items, and the TD group behaved similarly, shifting cutoff values for an average of 2.45 items (SD = 1.61, range 0–5), with no difference between groups (*t*(36) = 1.11, *p* = 0.276). With respect to the actual change in cutoff values, each emotion item (happy, sad, angry) demonstrated significantly shifted cutoff values from day 1 to day 2 (Happy: *t*(37) = −2.649, *p* = 0.012, Cohen’s *d* = −0.43; Sad: *t*(35) = =2.33, *p* = 0.032, Cohen’s *d* = −0.37; Angry: *t*(34) = −4.467, *p* < 0.001; Cohen’s *d* = −0.84) with no group differences (Happy: *t*(36) = 0.01, *p* = 0.994; Sad: *t*(34) = 0.05, *p* = 0.958; Angry: *t*(36) = 1.09, *p* = 0.284). The effect sizes of the shifts did vary, with the angry items showing the largest cutoff shifts and effect size (see Table 4 for cutoff values).

Our second research question asked how the participants’ general cutoff shift patterns might relate to their autistic traits as designated by their ADOS-2 total score, Social Communication score, and Restricted and Repetitive Behaviors score; how their pencil length cutoff shift patterns might relate to their cognitive levels as designated by their DAS scores; and how their emotion adjective cutoff shift patterns might relate to their language levels as designated by their CELF scores. Our regression analyses allowed us to see if relationships varied by diagnostic group. To examine whether cutoff shift scores were associated with autistic traits as measured by the *ADOS-2* and whether these associations differed by group, we conducted a series of regression models with interaction terms between group (TD vs. ASD) and test scores. Cutoff shift scores were not significantly related to *ADOS-2* outcomes in any model. The *ADOS-2* total score was not a significant predictor of Pencil Cutoff Shift (β = −0.03, 95% CI [−0.13, 0.07], *p* = 0.535) or Emotion Cutoff Shift (β = −0.01, 95% CI [−0.07, 0.05], *p* = 0.709). Similar patterns were observed for the Social Affect domain (Pencil: β = −0.03, *p* = 0.597; Emotion: β = −0.01, *p* = 0.722) and the Restricted and Repetitive Behaviors domain (Pencil: β = −0.13, *p* = 0.495; Emotion: β = −0.03, *p* = 0.779). No significant interaction terms were observed (all *p*s > 0.47), indicating that the relationship between cutoff shift and *ADOS-2* scores did not differ by group. Full model estimates are provided in Supplemental Table S1.

We next examined whether cutoff shifts were related to performance on nonverbal IQ and language measures. Models were constructed to determine if Pencil Cutoff Shift was associated with performance on *DAS-II* nonverbal composite scores and whether this association differed by group. Overall, Pencil Cutoff Shift was not consistently related to *DAS-II* performance. However, significant interactions were found for Nonverbal Reasoning Ability (β = 0.06, 95% CI [0.02, 0.10], *p* = 0.008, adjusted *p* = 0.019), Spatial Ability (β = 0.06, 95% CI [0.03, 0.10], *p* = 0.001, adjusted *p* = 0.004), and the Special Nonverbal Composite (β = 0.07, 95% CI [20.03, 0.10], *p* = 0.001, adjusted *p* = 0.004), indicating that the association between Pencil Cutoff Shift and performance differed by group. In each case, higher *DAS-II* scores were associated with a small (non-significant) decrease in pencil shift for the ASD group, whereas in the TD group, pencil shift increased with better performance on the *DAS-II*. Model *p*-values were adjusted for multiple comparisons (across nonverbal IQ and language measures) using the FDR method. Full model estimates are presented in Supplemental Table S2, and subtest models are presented in Supplemental Table S3.

We further examined whether Emotion Cutoff Shift was associated with performance on *CELF-5* and whether this relationship differed by group. We examined their total raw scores and Expressive Language Index, adjusting *p*-values for multiple comparisons using FDR. Overall, Emotion Cutoff Shift was not related to language performance, and no interactions were observed. Full model estimates and adjusted *p*-values are presented in Supplemental Table S4 and models containing CELF subtests in Table S5.

Given that the largest effect size was observed for angry faces, we examined whether Angry Cutoff Shift was specifically associated with *CELF-5* performance and whether these relationships differed by group. Significant associations of Angry Cutoff Shift were found for the *Total Raw Score* (β = −0.01, 95% CI [−0.02, −0.00], *p* = 0.025), with a significant group interaction (β = 0.03, 95% CI [0.00, 0.05], *p* = 0.019, adjusted *p* = 0.033). The positive interaction indicating that the relationship between Angry Cutoff Shift and language performance was attenuated for TD children compared to the ASD group. No relationships were seen for the Expressive Language Index. Full model estimates and adjusted *p*-values are presented in Supplemental Table S6, and models with CELF subtests can be found in Table S7.

**Figure 3:**
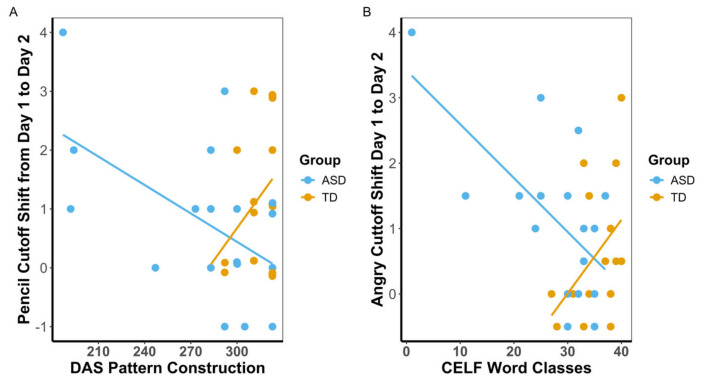
Scatterplots showing relationships between language (CELF) scores and cutoff shift from day 1 to day 2 for ‘Angry’ items (**A**) and between nonverbal IQ (DAS) and cutoff shifts for ‘Long’ items (**B**). Higher values on the Y-axis indicate a greater increase in the cutoff point from day 1 to day 2.

In general, participants with stronger nonverbal cognitive abilities in the ASD group demonstrated smaller cutoff shifts from day 1 to day 2 for the gradable adjective ‘long’, while for the TD group stronger nonverbal cognitive abilities were related to larger cutoff shifts. Similarly, participants with stronger language abilities, especially in the ASD group, demonstrated smaller cutoff shifts from day 1 to day 2, for the gradable adjective ‘angry.’ Figure 3 captures one of the DAS–Pencil Cutoff Shift relationships, and one of the CELF–Angry Cutoff Shift relationships, illustrating the interactions by group as well.

## 4. Discussion

For this study we asked two questions: First, would adolescent or young adult individuals who were on the autism spectrum, or were typically developing, shift their cutoff values for what counted as ‘long’, ‘happy’, ‘sad’, or ‘angry’ when more extreme examples of these physical or emotional dimensions were added to the stimulus set, and would these groups differ in their shifts? Our findings provide a resounding affirmative to the first part of this question and an equally resounding negative to the second part: Both groups shifted cutoff values for both pencils and emotions, almost to the same extent (Figure 2). Our second question asked the following: was the degree that the participants shifted their cutoff values related to their autistic characteristics, cognitive levels, and/or language levels? Our findings on this question are more complicated: unexpectedly, autistic characteristics as measured by the ADOS did not significantly relate to the size of shifts in cutoff values for either group, neither for physical nor emotional dimensions. Consistent with a possible role for spatial reasoning and/or acuity in our task, cognitive characteristics did relate significantly and positively to size of cutoff shifts for the pencil (‘long’) item and for the TD group. Consistent with a possible role for language in our task, language abilities related significantly to the size of cutoff shifts for the emotion items, although the only emotion item to reach significance was ‘angry’; unexpectedly, for the ASD group, the relationships were negative (Figure 3). In a nutshell, the relationships between cutoff shifts and cognitive and language abilities seemed to be different for the two groups: positive for the TD group, and less positive, or negative, for the ASD group.

Contrary to our main hypothesis, the individuals in the ASD group showed context sensitivity for both physical and emotion gradable adjectives; that is, they shifted their threshold of which pencils or faces counted as ‘long’, ‘angry’, ‘sad’, and ‘happy’ when given a preponderance of extremely long/angry/sad/happy items, and they did this as consistently as the individuals in the TD group. They did not shift their thresholds when given a preponderance of non-striped or non-spotted items, showing that they distinguished gradable adjectives and properties from absolute ones. These findings suggest that these individuals on the autism spectrum have at least basic command of this semantic/pragmatic distinction and so are demonstrating that their challenges with these linguistic domains are not pervasive. Indeed, a majority of these individuals have also manifested more complex categorical induction abilities than expected or than demonstrated when they were younger (Corrigan & Naigles, 2025). It is also particularly impressive that these autistic individuals demonstrated contextual sensitivity within the social domain of emotion understanding: on day 2, they were able to scrutinize the 13 faces showing variations in the emotions of happiness, sadness, or anger, recognize the preponderance of the extreme faces, and shift their thresholds of what counted as each of these emotions. Moreover, it is intriguing that their context sensitivity was reflected by visual adjustments in the stimuli. Whereas recent research with TD preschoolers has found young children to prioritize linguistic over visual information with physical gradable adjectives (Weicker & Schulz, 2024), the sensitivity of these autistic adolescents to small visual differences to distinguish emotional variability may reflect the attention to visual detail previously reported for individuals on the autism spectrum (Mottron et al., 2006).

The sensitivity to emotion context in the ASD group seems contrary to previous reports of autistic children’s challenges with emotion–word matching (Baron-Cohen et al., 2009; Golan et al., 2017; Taylor et al., 2015; Löytömäki et al., 2020). However, the participants in those earlier studies were primarily school-aged children rather than adolescents, and the largest differences with TD controls primarily involved mapping of faces to emotional tones of voice; in fact, some (e.g., Golan et al., 2017) found word–face matching to be similar in their ASD and TD groups. Moreover, Baron-Cohen et al. (2009) found that children on the autism spectrum showed markedly better performance with emotion understanding after targeted interventions; the autistic individuals in the current study were all adolescents or young adults, who have experienced a wide range of interventions at least through their school years, within which emotion understanding was likely included. Thus, possibly the context sensitivity of emotion adjective understanding is later-developing but not outside of the capacity of individuals with autism. Two additional points of note are that the emotions (and emotion words) targeted in the current study are among the first ones learned and are frequently used (Baron-Cohen et al., 2010); it remains to be seen if similar context sensitivity would be found with later-learned and lower frequency emotion words such as ‘disgust’ (Icht et al., 2021). Furthermore, the degree of cutoff shift observed in the current study, for both TD and ASD groups, was about half the size of that Britton et al. observed with their 4-year-old participants. It is possible there is a developmental pattern wherein older children remember their previous responses better and so become less guided or swayed by the new stimuli.

### 4.1. Relationships Between Gradable Adjective Understanding and Standardized Assessments

With respect to the autistic characteristics that might relate to gradable adjective interpretation, we expected a relationship to be observed with the restricted and repetitive behavior (RRB) component of the ADOS because RRBs might be reflective of context insensitivity and rigidity; this was not evident in our data. However, at both the Onset visit and the current one, the RRB subscores were quite low, ranging from 0 to 5 (whereas the social communication subscores (SC) were much higher, accounting for the bulk of the autism diagnosis and characteristics). Thus, the absence of a significant relationship might be attributed to the restricted range of the RRB scores; Troyb et al. (2014) also found that the ADI-R (Autism Diagnostic Interview-Revised; Lord et al., 1994), a parent report measure, was more sensitive to variability in restricted and repetitive behaviors across childhood, but this latter instrument was not administered to the full range of participants in the current study.

In contrast, the nonverbal-IQ assessment from the DAS-II yielded a significant difference in the relationships with the physical gradable adjective ‘long’ cutoff shifts between groups. For the DAS-II composites, neurotypical individuals who perceived spatial and/or visual pattern distinctions more accurately or had higher nonverbal cognitive functioning shifted their cutoffs of what counted as ‘long’ to a greater degree. That is, they restricted the ‘long’ box on day 2 to longer pencils than they had on day 1. We conjecture that these more spatially attuned individuals were the ones who more consistently noticed the changed preponderance of extremely long pencils on day 2, and this prompted them to change their ‘long’ thresholds. This pattern was not seen in our autistic participants. Because the DAS-II relationships included both nonverbal reasoning and spatial acuity subtests, we are unable to distinguish the roles of executive functions (including perspective taking) vs. visual/spatial focus; possibly, both are implicated in participants’ realizing how the adjusted context might sway their understanding of what counts as a long pencil.

For the Emotion Cutoff Shift scores in general, neither the total raw score nor the Expressive Language Index CELF-5 measures yielded a significant relationship. In contrast, with just the ‘angry’ face stimuli, overall CELF-5 raw scores, as well as many of the CELF subscales (word classes, formulated sentences, sentence assembly), yielded relationships with cutoff shift scores (see Tables S6 and S7). Interestingly, these were uniformly negative, especially for the ASD group. That is, the adolescents in this sample with more advanced language were *less* likely to adjust what counted as ‘angry’ on day 2, when four additional extremely angry faces were present. There are several non-overlapping reasons why this effect might have been observed only for ‘angry’. First, it is important to point out that while the ‘angry’ faces yielded on average the largest day 1–day 2 shift, there was no more variability for ‘angry’ than for ‘happy’ or ‘sad’ (see Table 4). Existing research suggests that angry faces are treated differently from, e.g., happy faces by children with ASD; for example, in eye tracking studies, Vacas et al. (2021) found that school age children with autism showed later orientation and less preference for angry faces than happy ones. Additionally, Wit et al. (2008) reported that 5-year-olds with ASD showed increased scanning of the eyes when viewing faces displaying negative emotions compared to those displaying positive emotions. Angry faces, then, might be considered more salient and thus might elicit less of the scrutiny that enabled the cutoff shifts. Ortony (2022), too, suggests that conditions of anger might be more homogeneous than those of joy or sadness—a little bit of anger could be just as easy to generate and/or just as aversive or noticeable as a lot of anger—thereby putting anger closer to the absolute adjective category.

The salience of anger, though, does not explain why the significant relationships observed, especially within the ASD group, with these faces were negative: why were the more advanced language users shifting less from day 1 to day 2, letting the additional ‘very angry’ faces influence their sorting less? It is hard to be certain, of course, but we speculate that these more cognitively and linguistically advanced teens were better at remembering their sorting from day 1, and especially with the more salient angry faces, just sticking to that for day 2. This may be where some autistic characteristics of these linguistically able adolescents were observed: Possibly, they thought to themselves, *I remember what I picked last time, I think I was correct last time, and I want to be consistent.* Some of our more reflective adolescents and young adults might also have been approaching Ortony’s (2022) suggestion that anger is closer to an absolute adjective and thus not shifted ‘on principle’ when the context changed. Conversely, those autistic teens with more modest language abilities behaved more like the younger children in Britton et al.’s (2017) study, with less meta-linguistic consideration of the day 1–day 2 contrast, and hence demonstrated more influence from the preponderance of very angry faces on day 2.

### 4.2. Limitations, Clinical Perspectives, and Future Directions

Some of this study’s limitations include its small sample size and the fact that the least verbal individuals with ASD in our original sample could not be included because we did not adapt the task to their level. Thus, it is unknown whether the flexibility (and lack thereof) we have reported will be demonstrated across the spectrum of autism, as well as with both these and more complex emotions. Moreover, it appears that the teens with lower language levels in the current sample might have performed differently, but the small number of low-language teens renders these relationships currently tentative. Still, our findings suggest that basic understanding of how interpretations of these three emotion words might be adjusted in different contexts is well within the reach of many adolescents with autism, and to the extent that this understanding was buttressed by interventions, we encourage such interventions to continue to be included in the treatment plans of children on the autism spectrum. Going forward, support for our interpretation of the negative relationships in the ASD group might have been strengthened had we asked the participants for justifications of their sortings, or recorded reaction times that indicated lengthier processing on day 2 by the more linguistically advanced participants. More detailed measures of autistic characteristics might also have revealed relationships with this index of language-related context sensitivity. Finally, additional tasks of context sensitivity with respect to gradable adjectives, as well as studies with speakers of non-English languages, would be useful to further explore the role of the size of the cutoff shifts, which might be reflective of the number and nature of the added stimuli as well as characteristics and native language of the participants.

## 5. Conclusions

This study revealed both similarities and differences between linguistically adept autistic and neurotypical adolescents and young adults. Both groups demonstrated the ability to distinguish gradable adjectives from absolute ones and to adjust their notions of what counts as ‘long’, ‘happy’, ‘sad’, and ‘angry’ when the contexts of the pencils (for ‘long’) and faces (for the emotions) were changed. Thus, the autistic group demonstrated an unexpected sensitivity to contextual perspectives. Interestingly, whereas the TD participants’ cognitive abilities generally positively predicted their degree of context sensitivity, the relationships observed with the autistic participants were generally negative. This suggests that the two groups were in some ways treating the task differently, and this might be reminiscent of findings from cortical neuroimaging, where similar language performance is distributed differently across the hemispheres in TD vs. ASD groups (e.g., Herringshaw et al., 2016). We encourage other researchers to further investigate.

## Figures and Tables

**Table 1:** Participant characteristics (M, SD) at the Onset visit.

Measure	TD (*n* =20)	ASD (*n* = 18)	*p*-Value
Age (months) *	18.75	32.00	<0.001
	(1.41)	(6.07)	
MSEL Expressive Language	18.35	19.11	0.71
	(5.92)	(6.45)	
MSEL Receptive Language	23.35	23.78	0.83
	(3.70)	(8.03)	
MSEL Visual Reception	24.75	26.61	0.25
	(4.48)	(5.37)	
ADOS-G total	1.55 (1.61)	15.50 (3.40)	<0.001

* MSEL = Mullen Scales of Early Learning; ADOS-G = Autism Diagnostic Observation Schedule-Generic; all scores are raw scores unless otherwise specified.

**Table 2:** Participant characteristics (M, SD) at the Outcome visit.

Measure	TD (*n* = 20)	ASD (*n* = 18)	*p*-Value
Age in years *	15.68	17.17	0.349
	(3.09)	(3.46)	
CELF-5 total	207.00	144.89	<0.001
	(21.18)	(58.39)	
CELF-5 LMI Standard score N in LMI in normal range (>/86)	107.10	77.89	<0.001
	(14.37)	(18.48)	
	20	7	
DAS-II total	68.45	50.11	<0.001
	(12.88)	(14.59)	
DAS-II SNC Standard score	107.70 (14.04)	81.11 (20.35)	<0.001
N in SNC normal range (>/85)	19	6	
ADOS-2 total	2.89 (3.25)	12.83 (6.95)	<0.001

* CELF-5 = Clinical Evaluation of Language Fundamentals—5th Edition; LMI = Language Memory Index; DAS-II = Differential Ability Scales—2nd Edition; SNC = Special Nonverbal Composite; ADOS-2 = Autism Diagnostic Observation Schedule—2nd Edition; all scores are raw scores unless otherwise specified. One TD participant is missing ADOS-2 data.

**Table 3:** Assessments administered at each visit.

Assessment *	Description	Subtests
	Onset Visit	
MSEL (Mullen, 1995)	NVIQ and expressive/receptive language assessment	Expressive Language, Receptive Language, Visual Reception
	Outcome Visit	
CELF-5 (Wiig et al., 2013)	Expressive/receptive Language assessment	Word Classes, Formulated Sentences, Recalling Sentences, Sentence Assembly, Semantic Relationships
DAS-II (Elliott, 2007)	NVIQ assessment	Recall of Designs, Pattern Construction, Matrices, Sequential and Quantitative Reasoning
ADOS-2 (Lord et al., 2000)	Autism Diagnosis and Characteristics	Social Communication, Restricted and Repetitive Behaviors

* MSEL = Mullen Scales of Early Learning; DAS = Differential Ability Scales; ADOS-2 = Autism Diagnostic Observation Schedule—2nd Edition; CELF-5 = Clinical Evaluation of Language Fundamentals—5th Edition; DAS-II = Differential Ability Scales—2nd Edition.

**Table 4:** Cutoff values (M, SD) on day 1 and day 2 by item type and group.

Item	TD (*n* = 20)	ASD (*n* = 18)
Spotted dog		
Day 1	5.0 (0)	5.0 (0)
Day 2	5.0 (0)	5.0 (0)
Striped Umbrella		
Day 1	5.0 (0)	4.83 (0.71)
Day 2	5.0 (0)	4.83 (0.79)
Long Pencils		
Day 1	5.92 (0.58)	5.97 (1.32)
Day 2	6.49 (0.54)	6.38 (0.48)
Emotional faces overall		
Day 1	4.88 (0.83)	4.75 (0.73)
Day 2	5.43 (1.34)	5.49 (0.92)
Happy Faces		
Day 1	5.65 (0.92)	5.44 (1.34)
Day 2	6.13 (1.41)	5.92 (1.57)
Sad Faces		
Day 1	4.20 (1.04)	4.17 (1.27)
Day 2	4.73 (1.54)	4.58 (1.43)
Angry Faces		
Day 1	4.80 (1.26)	4.14 (1.33)
Day 2	5.45 (1.57)	5.78 (1.09)

## Data Availability

Data from the Onset visit are in the process of being uploaded to Databrary.org. Data from the Outcome visit will be similarly uploaded when initial publications have been completed.

## Note

1 We include identity-first (*autistic person*) and person-first (*person on the autism spectrum*) language throughout this paper to reflect the variations in preference expressed by the autistic community (Bottema-Beutel et al., 2021); The first author identifies as neurodivergent; the second and third authors identify as neurotypical.

## Supplementary Materials

The following supporting information can be downloaded at: https://www.mdpi.com/article/10.3390/bs16020297/s1, Table S1: Regression analyses assessing the moderating role of Group in the association between Pencil Cutoff Shift and Emotion Cutoff Shift and ADOS-2; Table S2: Regression analyses assessing the moderating role of Group in the association between Pencil Cutoff Shift and DAS composites; Table S3: Regression analyses assessing the moderating role of Group in the association between Pencil Cutoff Shift and DAS subtests; Table S4: Regression analyses assessing the moderating role of Group in the association between Emotion Cutoff Shift and CELF-5; Table S5: Regression analyses assessing the moderating role of Group in the association between Emotion Cutoff Shift and CELF-5 subtests; Table S6: Regression analyses assessing the moderating role of Group in the association between Angry Cutoff Shift and CELF-5; Table S7: Regression analyses assessing the moderating role of Group in the association between Angry Cutoff Shift and CELF-5 subtests.

## Abbreviations

The following abbreviations are used in this manuscript:

MSEL	Mullen Scales of Early Learning
ADOS	Autism Diagnostic Observation Schedule
CELF	Clinical Evaluation of Language Fundamentals
NVIQ	Nonverbal Intelligence Quotient
ASD	Autism Spectrum Disorder
DAS	Differential Ability Scales
RRB	Restricted and Repetitive Behaviors
TD	Typically Developing
