# Supplementary material for: Autistic Individuals Are Flexible with Physical and Emotion Gradable Adjectives
Source: Behav Sci (Basel). 2026 Feb 19;16(2):297. doi: 10.3390/bs16020297 (PMC12938400; doi:10.3390/bs16020297)
Supplement: Supplementary file 1 [file behavsci-16-00297-s001.zip › behavsci-3934560-supplementary.pdf]

Table S1 Regression analyses assessing the moderating role of Group in the association between Pencil Cutoff Shift and Emotion Cutoff Shift and ADOS-2.

| ***Predictor*** | ***Estimate*** | ***95% CI*** | ***p*** |
|---|---|---|---|
| | **Emotion Cutoff Shift** | | |
| (Intercept) | 0.84 | -0.06 – 1.74 | 0.067 |
| **ADOS Total Score** | -0.01 | -0.07 – 0.05 | 0.709 |
| Group: TD | -0.08 | -1.13 – 0.97 | 0.881 |
| **ADOS Total Score** × Group: TD | -0.05 | -0.18 – 0.09 | 0.478 |
| (Intercept) | 0.85 | -0.12 – 1.81 | 0.084 |
| **Social Affect Domain Raw Score** | -0.01 | -0.09 – 0.06 | 0.722 |
| Group: TD | -0.09 | -1.20 – 1.01 | 0.865 |
| **SAD** × Group: TD | -0.05 | -0.20 – 0.10 | 0.522 |
| (Intercept) | 0.73 | 0.23 – 1.22 | **0.005** |
| **Restricted and Repetitive Behaviors Domain Raw Score** | -0.03 | -0.27 – 0.21 | 0.779 |
| Group: TD | -0.09 | -0.74 – 0.55 | 0.775 |
| **RRB** × Group: TD | -0.19 | -0.85 – 0.47 | 0.561 |
| | **Pencil Cutoff Shift** | | |
| (Intercept) | 1.16 | -0.27 – 2.58 | 0.109 |
| **ADOS Total Score** | -0.03 | -0.13 – 0.07 | 0.535 |
| Group: TD | 0.03 | -1.63 – 1.68 | 0.974 |
| **ADOS Total Score** × Group: TD | 0.05 | -0.16 – 0.26 | 0.623 |
| (Intercept) | 1.13 | -0.40 – 2.66 | 0.143 |
| **Social Affect Domain Raw Score** | -0.03 | -0.14 – 0.08 | 0.597 |
| Group: TD | 0.09 | -1.65 – 1.84 | 0.914 |
| **SAD** × Group: TD | 0.04 | -0.20 – 0.28 | 0.731 |
| (Intercept) | 0.91 | 0.14 – 1.68 | **0.023** |
| **Restricted and Repetitive Behaviors Domain Raw Score** | -0.13 | -0.50 – 0.25 | 0.495 |
| Group: TD | 0.26 | -0.75 – 1.26 | 0.608 |
| **RRB** × Group: TD | 0.48 | -0.55 – 1.51 | 0.349 |

*note:* ADOS-2-*Autism Diagnostic Observation Schedule-Second Edition* (Lord et al, 2012).

*Table S2* Regression analyses assessing the moderating role of Group in the association between Pencil Cutoff Shift and DAS composites.

| ***Predictor*** | ***Estimate*** | ***95% CI*** | ***p*** | **Adjusted *p*** |
|---|---|---|---|---|
| **Nonverbal Reasoning Ability** | | | | |
| (Intercept) | 2.31 | -0.29 – 4.91 | 0.080 | |
| NRA | -0.02 | -0.05 – 0.01 | 0.172 | |
| Group: TD | -5.69 | -10.03 – -1.35 | **0.012** | |
| NRA × Group: TD | 0.06 | 0.02 – 0.10 | **0.008** | **.019** |
| $R^2$ / $R^2$ adjusted: 0.156 / 0.084 | | | | |
| **Spatial Ability** | | | | |
| (Intercept) | 1.94 | -0.23 – 4.10 | 0.078 | |
| SA | -0.02 | -0.04 – 0.01 | 0.220 | |
| Group: TD | -5.71 | -8.97 – -2.46 | **0.001** | |
| SA × Group: TD | 0.06 | 0.03 – 0.10 | **0.001** | **.004** |
| $R^2$ / $R^2$ adjusted: 0.192 / 0.122 | | | | |
| **Special Nonverbal Composite** | | | | |
| (Intercept) | 3.09 | 0.30 – 5.88 | **0.031** | |
| SNC | -0.03 | -0.06 – 0.00 | 0.075 | |
| Group: TD | -6.84 | -10.52 – -3.17 | **0.001** | |
| SNC × Group: TD | 0.07 | 0.03 – 0.11 | **0.001** | **.004** |
| $R^2$ / $R^2$ adjusted: 0.238 / 0.172 | | | | |

*note*: DAS- *Differential Ability Scales*

*Table S3* Regression analyses assessing the moderating role of Group in the association between Pencil Cutoff Shift and DAS subtests.

| ***Predictor*** | ***Estimate*** | ***95% CI*** | ***p*** |
|---|---|---|---|
| **Pattern Construction** | | | |
| (Intercept) | 5.10 | 0.85 – 9.35 | **0.020** |
| PC | -0.02 | -0.03 – -0.00 | **0.036** |
| Group: TD | -15.46 | -25.38 – -5.55 | **0.003** |
| PC × Group: TD | 0.05 | 0.02 – 0.09 | **0.003** |
| $R^2$ / $R^2$ adjusted: 0.217 / 0.150 | | | |
| **Sequential and Quantitative Reasoning** | | | |
| (Intercept) | 3.01 | 1.15 – 4.87 | **0.002** |
| SQR | -0.02 | -0.03 – -0.00 | **0.010** |
| Group: TD | -1.94 | -7.61 – 3.72 | 0.490 |
| SQR × Group: TD | 0.02 | -0.02 – 0.05 | 0.313 |
| $R^2$ / $R^2$ adjusted: 0.169 / 0.098 | | | |
| **Recall of Design** | | | |
| (Intercept) | 2.56 | 0.00 – 5.11 | **0.050** |
| RD | -0.02 | -0.05 – 0.01 | 0.129 |
| Group: TD | -2.30 | -6.16 – 1.56 | 0.234 |
| RD × Group: TD | 0.03 | -0.01 – 0.07 | 0.125 |
| $R^2$ / $R^2$ adjusted: 0.077 / -0.002 | | | |
| **Matrices** | | | |
| (Intercept) | 2.87 | 0.27 – 5.47 | **0.031** |
| M | -0.02 | -0.05 – 0.00 | 0.075 |
| Group: TD | -2.61 | -7.36 – 2.14 | 0.272 |
| M× Group: TD | 0.03 | -0.01 – 0.07 | 0.167 |
| $R^2$ / $R^2$ adjusted: 0.100 / 0.023 | | | |

*note*: DAS- *Differential Ability Scales*

*Table S4* Regression analyses assessing the moderating role of Group in the association between Emotion Cutoff Shift and CELF-5.

| ***Predictor*** | ***Estimate*** | ***95% CI*** | ***p*** | **Adjusted *p*** |
|---|---|---|---|---|
| **Total Raw Score** | | | | |
| (Intercept) | 0.35 | -0.95 – 1.66 | 0.588 | |
| **Total** | 0.00 | -0.01 – 0.01 | 0.516 | |
| Group: TD | 0.23 | -2.86 – 3.31 | 0.883 | |
| **Total** × Group: TD | -0.00 | -0.02 – 0.01 | 0.713 | .713 |
| $R^2$ / $R^2$ adjusted: 0.032 / -0.054 | | | | |
| **Expressive Language Index** | | | | |
| (Intercept) | 0.39 | -1.52 – 2.30 | 0.681 | |
| ELI | 0.00 | -0.02 – 0.03 | 0.705 | |
| Group: TD | 1.69 | -1.54 – 4.92 | 0.294 | |
| ELI× Group: TD | -0.02 | -0.05 – 0.01 | 0.246 | .324 |
| $R^2$ / $R^2$ adjusted: 0.060 / -0.023 | | | | |

*note*: CELF-5- *Clinical evaluation of language fundamentals–fifth edition* (Wiig et al., 2013)

*Table S5* Regression analyses assessing the moderating role of Group in the association between Emotion Cutoff Shift and CELF-5 subtests.

| ***Predictor*** | ***Estimate*** | ***95% CI*** | ***p*** |
|---|---|---|---|
| **Word Classes** | | | |
| (Intercept) | 0.52 | -0.76 – 1.81 | 0.412 |
| WC | 0.01 | -0.04 – 0.05 | 0.745 |
| Group: TD | -2.01 | -5.06 – 1.04 | 0.189 |
| WC × Group: TD | 0.05 | -0.04 – 0.14 | 0.287 |
| **Formulated Sentences** | | | |
| (Intercept) | 0.44 | -0.94 – 1.82 | 0.520 |
| FS | 0.01 | -0.03 – 0.05 | 0.642 |
| Group: TD | 0.29 | -2.78 – 3.35 | 0.851 |
| FS × Group: TD | -0.01 | -0.09 – 0.06 | 0.706 |
| **Recalling Sentences** | | | |
| (Intercept) | 0.20 | -0.98 – 1.38 | 0.733 |
| RS | 0.01 | -0.01 – 0.03 | 0.298 |
| Group: TD | -0.83 | -4.76 – 3.10 | 0.670 |
| RS × Group: TD | 0.00 | -0.05 – 0.06 | 0.863 |
| **Sentence Assembly** | | | |
| (Intercept) | 0.32 | -0.77 – 1.41 | 0.553 |
| SA | 0.03 | -0.04 – 0.11 | 0.338 |
| Group: TD | 0.60 | -0.82 – 2.03 | 0.394 |
| SA × Group: TD | -0.07 | -0.17 – 0.04 | 0.202 |
| **Semantic Relationships** | | | |
| (Intercept) | 0.59 | -0.14 – 1.32 | 0.110 |
| SR | 0.02 | -0.05 – 0.08 | 0.609 |
| Group: TD | 0.35 | -0.57 – 1.26 | 0.446 |
| SR × Group: TD | -0.04 | -0.13 – 0.04 | 0.274 |

*note*: CELF-5- *Clinical evaluation of language fundamentals–fifth edition* (Wiig et al., 2013)

*Table S6* Regression analyses assessing the moderating role of Group in the association between Angry Cutoff Shift and CELF-5.

| ***Predictor*** | ***Estimate*** | ***95% CI*** | ***p*** | **Adjusted *p*** |
|---|---|---|---|---|
| **Total Raw Score** | | | | |
| (Intercept) | 2.85 | 1.21 – 4.49 | **0.001** | |
| **Total** | -0.01 | -0.02 – -0.00 | **0.025** | |
| Group: TD | -5.16 | -9.28 – -1.05 | **0.016** | |
| **Total** × Group: TD | 0.03 | 0.00 – 0.05 | **0.019** | **.033** |
| $R^2$ / $R^2$ adjusted: 0.243 / 0.176 | | | | |
| **Expressive Language Index** | | | | |
| (Intercept) | 3.63 | 0.70 – 6.55 | **0.017** | |
| ELI | -0.03 | -0.07 – 0.00 | 0.067 | |
| Group: TD | -2.59 | -7.48 – 2.31 | 0.290 | |
| ELI× Group: TD | 0.03 | -0.02 – 0.08 | 0.278 | .324 |
| $R^2$ / $R^2$ adjusted: 0.158 / 0.083 | | | | |

*note*: CELF-5- *Clinical evaluation of language fundamentals–fifth edition* (Wiig et al., 2013)

*Table S7* Regression analyses assessing the moderating role of Group in the association between Angry Cutoff Shift and CELF-5 subtests.

| ***Predictors*** | ***Estimates*** | ***CI*** | ***p*** |
|---|---|---|---|
| **Word Classes** | | | |
| (Intercept) | 3.42 | 2.07 – 4.77 | **<0.001** |
| WC | -0.08 | -0.13 – -0.04 | **0.001** |
| Group: TD | -6.66 | -9.92 – -3.40 | **<0.001** |
| WC × Group: TD | 0.19 | 0.09 – 0.29 | **<0.001** |
| **Formulated Sentences** | | | |
| (Intercept) | 2.80 | 1.04 – 4.56 | **0.003** |
| FS | -0.05 | -0.10 – -0.00 | **0.039** |
| Group: TD | -4.95 | -8.00 – -1.90 | **0.002** |
| FS × Group: TD | 0.12 | 0.04 – 0.20 | **0.005** |
| **Recalling Sentences** | | | |
| (Intercept) | 2.61 | 1.03 – 4.20 | **0.002** |
| RS | -0.03 | -0.06 – -0.00 | **0.023** |
| Group: TD | -6.66 | -11.26 – -2.06 | **0.006** |
| RS × Group: TD | 0.10 | 0.03 – 0.17 | **0.008** |
| **Sentence Assembly** | | | |
| (Intercept) | 2.46 | 1.19 – 3.73 | **<0.001** |
| SA | -0.12 | -0.21 – -0.03 | **0.011** |
| Group: TD | -1.06 | -3.46 – 1.34 | 0.377 |
| SA × Group: TD | 0.06 | -0.09 – 0.22 | 0.415 |
| **Semantic Relationships** | | | |
| (Intercept) | 1.16 | 0.15 – 2.17 | **0.026** |
| SR | -0.01 | -0.11 – 0.08 | 0.779 |
| Group: TD | -1.14 | -3.23 – 0.94 | 0.274 |
| SR × Group: TD | 0.05 | -0.10 – 0.19 | 0.503 |

*note*: CELF-5- *Clinical evaluation of language fundamentals–fifth edition* (Wiig et al., 2013)
